# Distinct gene expression profiles associated with the susceptibility of pathogen-specific CD4+ T cells to HIV-1 infection

**DOI:** 10.1186/1742-4690-9-S2-O46

**Published:** 2012-09-13

**Authors:** H Hu, M Nau, P Ehrenberg, A Chenine, Z Daye, Z Wei, N Michael, M Vahey, J Kim, M Marovich, S Ratto-Kim

**Affiliations:** 1 U.S. Military HIV Research Program, Silver Spring, MD, USA; 2 University of Pennsylvania School of Medicine, Philadelphia, PA, USA; 3 New Jersey Institute of Technology, Newark, NJ, USA

## Background

HIV infection causes the progressive depletion of CD4+ T cells. Contrary to the early loss of CD4 response to opportunistic pathogens like Candida albicans, cytomegalovirus (CMV)-specific CD4 response is persistent when total CD4+ T cell number is low. The mechanism is less clear. Despite considerable knowledge for the impact of HIV infection on total CD4+ T cells and their subsets, little is known about HIV infection of CD4+ T cells of different pathogen/antigen (Ag) specificity.

## Methods

PBMC from HIV-negative donors were CFSE-labeled and stimulated ex vivo with pathogen-specific antigens including viral (CMV), bacterial (Tetanus Toxoid: TT) and fungal (Candida albicans) antigens. HIV infection of Ag-specific CD4+ T cells was determined by intracellular p24 production in CFSE-low population.

## Results

While TT- and Candida-specific CD4+ T cells were permissive, CMV-specific CD4+ T cells are highly resistant to both X4 and R5 HIV independent of coreceptor useage. Quantification of HIV DNA in sorted, antigen-specific CD4+ T cells demonstrated a reduction of both strong-stop and full-length HIV DNA in CMV-specific CD4+ T cells. β-chemokine neutralization enhanced HIV entry and viral replication in TT- and Candida-specific CD4+ T cells, whereas HIV infection in CMV-specific CD4+ T cells remained low despite increased HIV entry by β-chemokine neutralization, suggesting both entry and post-entry HIV restriction in CMV-specific cells. Microarray analysis revealed distinct gene expression profiles that involved selective upregulation of a broad array of antiviral genes in CMV-specific CD4+ T cells, whereas TT- and Candida-specific CD4+ T cells mainly upregulated a Th17 inflammatory response.

## Conclusion

Our data suggest a mechanism for the persistence of CMV-specific CD4 response and the earlier loss of mucosal Th17-associated TT- and Candida-specific CD4 response in AIDS patients. The model described is useful in HIV vaccine studies by evaluating the susceptibility of vaccine-specific CD4 responses to HIV infection.

